# Viruses in Horses with Neurologic and Respiratory Diseases

**DOI:** 10.3390/v11100942

**Published:** 2019-10-14

**Authors:** Eda Altan, Yanpeng Li, Gilberto Sabino-Santos Jr, Vorthon Sawaswong, Samantha Barnum, Nicola Pusterla, Xutao Deng, Eric Delwart

**Affiliations:** 1 Vitalant Research Institute, San Francisco, CA 94118, USA; EAltan@vitalant.org (E.A.); YLi@vitalant.org (Y.L.); GSabino-SantosJr@vitalant.org (G.S.-S.J.); vorthon007.giftedcru@gmail.com (V.S.); XDeng@vitalant.org (X.D.); 2 Department of Laboratory Medicine, University of California, San Francisco, CA 94118, USA; 3 Department of Medicine and Epidemiology, School of Veterinary Medicine, University of California, Davis, CA 95616, USA; smmapes@ucdavis.edu (S.B.); npusterla@ucdavis.edu (N.P.)

**Keywords:** *Parvoviridae*, *Eqcopivirus*, horse parvovirus-CSF, equine hepacivirus, equine parvovirus H, bosavirus, virome

## Abstract

Metagenomics was used to identify viral sequences in the plasma and CSF (cerobrospinal fluid) of 13 horses with unexplained neurological signs and in the plasma and respiratory swabs of 14 horses with unexplained respiratory signs. Equine hepacivirus and two copiparvoviruses (horse parvovirus-CSF and a novel parvovirus) were detected in plasma from neurological cases. Plasma from horses with respiratory signs contained the same two copiparvoviruses plus equine pegivirus D and respiratory swabs contained equine herpes virus 2 and 5. Based on genetic distances the novel copiparvovirus qualified as a member of a new parvovirus species we named *Eqcopivirus*. These samples plus another 41 plasma samples from healthy horses were tested by real-time PCRs for multiple equine parvoviruses and hepacivirus. Over half the samples tested were positive for one to three viruses with eqcopivirus DNA detected in 20.5%, equine hepacivirus RNA and equine parvovirus-H DNA in 16% each, and horse parvovirus-CSF DNA in 12% of horses. Comparing viral prevalence in plasma none of the now three genetically characterized equine parvoviruses (all in the copiparvovirus genus) was significantly associated with neurological and respiratory signs in this limited sampling.

## 1. Introduction

The United States has the largest horse population with 9.5 million horses, followed by those of China, Mexico, Brazil, and Argentina with an estimated 2006 world population of 68 million [1].

Equine viral pathogens belonging to diverse viral families have been described including, but not limited to, equid herpesviruses, equine arteritis virus, African horse sickness virus, equine infectious anemia virus, equine coronavirus, Hendra virus, vesicular stomatitis virus, equine influenza virus, West Nile virus, Eastern Equine Encephalitis, Venezuelan Equine Encephalitis, Western Equine Encephalitis, and most recently equine parvovirus-H causing hepatitis [2,3,4,5,6,7,8,9].

A limited number of viral metagenomics studies have analyzed horse samples. A 2013 study identified a novel flavivirus that was named Theiler’s disease-associated virus (TDAV) from an outbreak of this liver disease transmitted through equine-origin tetanus anti-toxin [10]. Equine parvovirus-H in the copiparvovirus genera (EqPV-H) was first described in 2018 [2] and was recently shown through epidemiological studies and inoculation studies to be a more likely cause of Theiler’s disease caused either by transfusion of contaminated horse serum or through naturally acquired infections (although asymptomatic infections are also frequent) [11,12,13]. A hepacivirus closely related to HCV (hepatitis C virus), initially characterized by metagenomics from a dog [14], but subsequently found to more frequently infect horses was named equine hepacivirus (EqHV) but was not associated with serum hepatitis [15,16,17]. Equine pegivirus (EPgV), most closely related to a bat pegiviruses [18], was first identified using a degenerate PCR approach [19] and shown to be a common equine infection but was also not associated with equine liver disease [15]. 

Beside the above studies focused on Theiler’s disease viral metagenomics study of CSF from a horse with neurological signs identified another parvovirus named horse parvovirus-CSF [20]. Metagenomics studies of equine serum pools recently identified the four equine viruses listed above plus unexpectedly the porcine Suid betaherpesvirus 2 [21]. 

To further characterize the eukaryotic virome of horses and identify possible equine pathogens we used here a combination of viral metagenomics and real-time PCR to analyze plasma and cerebrospinal fluid (CSF) from 13 horses with unexplained neurological signs, plasma and respiratory swabs from 14 horses with unexplained respiratory signs, and plasma from 41 healthy horses, when necessary.

## 2. Materials and Methods 

### 2.1. Study Samples

The use of horse samples adhered to the animal use guidelines set by UC Davis’ Institutional Animal Care and Use Committee (AICUC Protocol # 19988, approval date 5/31/2018).

In this study 68 horses were analyzed using one or two of three types of biological samples (plasma, CSF, and respiratory swabs). Both plasma and CSF were collected from 13 horses that showed acute neurological signs (behavioral changes, spinal ataxia, proprioceptive deficits and/or cranial nerve deficits). Plasma and respiratory swabs were collected from another 14 horses with fever (>38.6 °C) and nasal discharge/cough (the respiratory swab from animal 5 is missing). Plasma was also collected from 41 healthy horses. 

The 13 horses with neurological signs each had CSF with evidence of lymphocytic pleocytosis, negative antibody titters to *Sarcocysits neurona* and *Neospora hughesi* by immunodiagnostics (indirect fluorescence antibody tests) and no detection of *S. neurona*, *N. hughesi*, and *Borrelia burgdorferi* by PCR. The 13 horses with respiratory signs each had respiratory swabs that tested non-reactive for EHV-1/-4, EIV, ERAV/ERBV, and *Streptococcus equi* subspecies *equi*.

All 53 samples from these 27 sick horses were analyzed in 12 pools of 4–5 samples using viral metagenomics (Table 1).

### 2.2. Viral Metagenomics

For viral metagenomics plasma, CSF, and respiratory swab samples were clarified by 14,000 rpm centrifugation for five minutes, and filtered using a 0.45-µm filter (Merck Millipore Ltd., Cork, Ireland). Free nucleic acids in the 400 µL filtrates were digested using DNAse and RNAse enzymes to enrich for viral nucleic acids protected within viral capsids. Nucleic acids were then extracted (MagMAX Viral RNA Isolation Kit, Ambion, Inc, Austin, TX, USA) [22] and amplified by random RT-PCR followed by use of the Nextera™ XT Sample Preparation Kit (Illumina) to generate a library for Illumina MiSeq (2 × 250 bases) with dual barcoding as previously described [23]. 

An in-house analysis pipeline was used to analyze sequence data. Before analyzing, raw data were pre-processed by subtracting human and bacterial sequences, duplicate sequences, and low quality reads. Following de novo assembly using the Ensemble program [24], both contigs and singlets viral sequences were then analyzed using translated protein sequence similarity search (BLASTx v.2.2.7) to all annotated viral proteins available in GenBank. Candidate viral hits were then compared to an in-house non-virus non-redundant (nr) protein database to remove false positive viral hits. To align reads and contigs to reference viral genomes from GenBank and generate complete or partial genome sequences the Geneious R10 program was used.

### 2.3. Generation of Full Genomes of Novel Horse Parvovirus

Following mini-pool metagenomics sequencing three individual samples were re-sequenced using the same method to generate 3 near full genomes (complete ORFs) of eqcopiviruses (EqCoPV) (deposited in GenBank with accession numbers MN181466-8 for eqcopivirus 8, 9, and 11 derived from respiratory symptoms plasma samples 4, 5, and 7 respectively). Genome gaps left by high throughput sequencing (HTS) were filled by PCR and products were Sanger sequenced.

### 2.4. Real-Time PCR

Real-time PCR assays were developed to the three parvoviruses detected here using HTS (horse parvovirus-CSF, novel eqcopivirus, and bovine serum-associated bosavirus). Real-time PCR assays were also designed for equine hepaciviruses detected in one plasma pool and for the recently identified hepatotropic equine parvovirus-H (EqPV-H) pathogen using previously described PCR conditions [12,17]. Real-time PCR amplification was performed in a Roche 480 thermocycler. Primers and probes were used in multiplex real-time PCR (eqcopivirus, horse parvovirus-CSF, and bosavirus) and a separate multiplex real-time PCR for equine hepacivirus and EqPV-H. Primer, probes, oligonucleotide sequences, and length of amplicons are summarized in Supplementary Table S1. The reaction mix for each sample consisted of 12.5 µL QuantiFast Probe PCR Master Mix (Qiagen, Hilden, Germany), 1M each primer, 0.5 M each probe, and 3 µL DNA or cDNA. Real-time PCR sensitivity was first determined using known concentrations of oligonucleotides representing the PCR target regions. Oligonucleotides were serially diluted and real-time PCR results endpoint detection were estimated at 100 to 125 input genome equivalents.

### 2.5. Phylogenetic Analysis

The NS1 and VP1 protein sequences of parvoviruses were aligned using Clustal W in Geneious v10.1.3. and phylogenetic trees constructed using the Maximum likelihood method with two substitution models: Le_Gascule_2008 model (LG) with Freqs and gamma distributed, invariant sites (G + I) MEGA software ver. X [25]. The substitution models were selected based on the results of the Best Model search of MEGA X. The percentage of trees in which the associated taxa clustered together is shown next to the branch points. Initial tree(s) for the heuristic search were obtained by applying the neighbor-joining method to a matrix of pairwise distances estimated using the maximum composite likelihood (MCL) approach. The tree is drawn to scale, with branch lengths measured in the number of substitutions per site. The phylogenetic analysis used sequences from nineteen different copiparvoviruses and human parvovirus B19 (AY386330) was used to root the tree.

## 3. Results

### 3.1. Viral Metagenomics

Plasma and CSF from 13 horses with neurological signs and plasma and respiratory swabs from another 14 horses with respiratory signs tested negative for a panel of known equine pathogens (see Study Samples in Materials and Methods). 

These samples were analyzed by viral metagenomics in 12 pools of 4–5 samples each. A total of 15.2 million reads were generated for an average number of reads of ~1.1 million per pool. The raw sequence data for each pool are available at NCBI*’*s Short Reads Archive under GenBank accession number SRP120619.

Viruses detected with BLASTx translated protein matches (E score <10^−10^) were: a novel copiparvovirus in three pools, bovine serum-associated parvovirus (bosavirus) in three pools, horse parvovirus-CSF in two pools, and equine hepacivirus, equine pegivirus D, and equid gammaherpesvirus 2 and 5 in one pool each (Table 1).

The most commonly detected eukaryotic viral reads belonged to the *Copiparvovirus* genus of *Parvoviridae* family with a frequency ranging from 0.66% to 3.68% of total reads. Novel (divergent) copiparvovirus reads from three pools generated contigs ranging in size from 1417 to 5158 nt that showed closest translated aa identity of 42.5 to 43.9% to NS1 and 43.5 to 44.9% to VP1 encoded by the horse parvovirus-CSF genome (GenBank KR902500). This virus was named equine copiparvovirus (eqcopivirus or EqCoPV).

Reads matching the previously described horse parvovirus-CSF genome were also detected. This parvovirus was reported in the CSF of a horse with neurological signs [20].. A partial NS1 sequence of this virus was also recently reported from a thoroughbred in China sampled in 2018 (QCF41227.1). Two of the three pools containing the novel eqcopivirus also showed the presence of horse parvovirus-CSF although with fewer reads. Both 37.5% and 60.5% of the horse parvovirus-CSF genomes could be assembled using reads from these plasma pools showing 98.8% and 97% nucleotide similarity with the original horse parvovirus-CSF derived genome in GenBank (KR902500).

Equine hepacivirus contigs of 522 and 657 nucleotides were also generated from seven reads in a pool of plasma from horses with neurological signs. Contigs showed 97.9–98.7% nt identities to equine hepacivirus (JQ434008) in GenBank.

A total of 13 different equid gammaherpesvirus 2 and 5 contigs, ranging in size from 446 to 1049 nucleotides, were generated from 120 and 25 reads in a pool of swab from horses with respiratory symptom. Contigs showed 92–100% aa identities to equid gammaherpesvirus 2 (NC_001650.2) and 5 (NC_026421.1) in GenBank.

Two partial equine pegivirus contigs of 446 and 903 nucleotides were generated from 317 reads in a pool of plasma from horses with respiratory signs. Contigs showed 92–93% nt identities to equine pegivirus 1 (KC410872) in GenBank.

Another copiparvovirus (bosavirus) was also detected but likely originated from fetal bovine Plasma spiked into the respiratory swab viral transport medium. A near complete genome from a respiratory swab pool showed 100% aa identity to that of bosavirus in GenBank (NC_031959) [26].

### 3.2. Generation of Near-Full Length Genomes of Novel Equine Copiparvovirus (Eqcopivirus)

The length of the longest eqcopivirus contig was 5159 nucleotides (nt) with typical copiparvovirus genome organization of two complete major ORFs (NS and VP), and 5’ and 3’ UTRs missing the genome’s terminal hairpin sequences. The study strain had a 43.8% G+C content and it has a nt distribution of 39.3% A, 16.8% T, 22.8% G, and 20.9% C. 

The expected NS1 ATP- or GTP-binding Walker A loop motif (GxxxxGKT/S; GPPSVGKS) and Walker B motif (EE) were all found [27,28]. The phospholipase A2 (PLA2) catalytic residues (HDLGY) and its highly conserved calcium-binding site (YTGPG) were also found at the *N*-terminus of the capsid protein [29].

**Figure 1:**
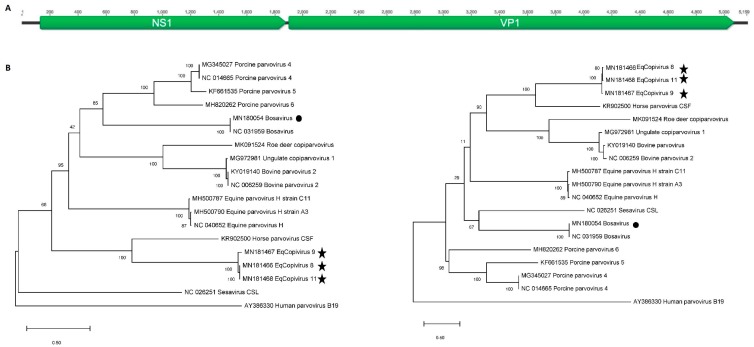
(**A**) Genome ORF (Open Reading Frame) structure of eqcopivirus genome. (**B**) Phylogenetic analysis of NS1 (left) and VP1 (right) proteins in the *Copiparvovirus* genus. Bootstrap values from a hundred replicate runs are shown. Symbols are used to highlight the new genomes described here.

The predicted capsid proteins (VP1) of eqcopivirus showed closest aa identity of 43.4% (coverage: 77%) and 37.7% (coverage: 53%) to the corresponding proteins of its two closest relative the horse parvovirus-CSF (KR902500) and equine parvovirus-H (MG136722), respectively. The nonstructural protein (NS1) proteins had 43.4% (coverage: 74%) and 31.3% (coverage: 69%) aa identity to the corresponding proteins of horse parvovirus-CSF (KR902500) and equine parvovirus-H (MG136722), respectively. The ORF structure of eqcopivirus is shown in Figure 1A. Phylogenetic analyses of the eqcopiviruses and bosavirus NS and VP proteins acquired in this study are shown in Figure 1B.

### 3.3. Real-Time PCR Results

Real-time PCR assays were developed to the three parvoviruses detected here using HTS (horse parvovirus-CSF, novel eqcopivirus, and bosavirus). Real-time PCR assays using previously described conditions were used for equine hepaciviruses and the recently identified hepatotropic equine parvovirus-H pathogen [12,19]. These PCR assays were then used on all 94 available equine plasma, CSF, and respiratory swabs (from 68 horses) described above. The positive detections are shown with Ct values that are inversely proportional to their nucleic acid target concentration (Table 2).

Bosavirus (originally reported in bovine serum pools) [26] was detected in every one of the 13 respiratory swab samples with nearly identical C*_T_* value. These respiratory swabs had been preserved in a transport media spiked with fetal bovine serum from the same commercial product. None of the other equine samples (plasma and CSF) were preserved using fetal bovine serum and none showed the presence of bosavirus parvovirus DNA by HTS or real-time PCR. Because bosavirus was originally described as a contaminant of fetal bovine serum [26] we ascribe its detection to the use of contaminated fetal bovine serum spiked into every respiratory swab sample.

The most commonly detected virus was the new eqcopivirus detected in 16/94 samples (14/68 horses). Its highest detection rate was in 4/14 plasmas from respiratory cases. Three of the four plasma positive samples also had matching respiratory swabs, two of which were also positive indicating the presence of eqcopivirus genomes in both plasma and respiratory swabs and revealing a possible mode of transmission through respiratory fluids (Table 2). When the eqcopivirus prevalence in plasma samples from respiratory cases (4/14 or 28.6%) was compared to that in plasma from healthy animals (7/41 or 17%) the difference (measured using Fisher’s exact test) yielded a non-significant p value of 0.443 (Table 3). The next two most prevalent viruses were the hepatotropic parvovirus-H and equine hepacivirus both found in 11/89 samples including 3 co-infections. All detections were in plasma samples except for two hepacivirus positive respiratory swabs (Table 2). No evidence of higher virus prevalence was detected between cases and health controls (Table 3). Lastly the horse parvovirus-CSF was detected in eight samples from eight horses in all sample types (plasma, CSF, respiratory swabs) also with no obvious association with either neurological or respiratory disease when comparing prevalence in plasma samples (Table 3). When the real-time PCR Ct values were compared (using unpaired two tailed T-test with Welsh’s correction) no statistically significant differences were found between cases and controls.

## 4. Discussion

Virus metagenomics analysis of plasma, CSF and respiratory swabs from horses with unexplained neurological and respiratory signs showed the presence of three parvoviruses, one of which (bosavirus) was likely introduced by the addition of fetal bovine serum to the respiratory swabs transport medium. Additionally detected by metagenomics were equine hepacivirus, equine pegivirus [19], and equine herpesvirus 2 and 5. Another copiparvovirus (equine parvovirus-H) was also detected using real-time PCR (but not by HTS) likely due to its lower concentration as reflected by higher real-time PCR C*_T_* values. 

The presence of viral nucleic acids detected by PCR or HTS does not prove the presence of infectious viruses. Nonetheless, the repeated detection of viral genomes in nuclease-treated, presumably sterile samples such as plasma and CSF, and the typically rapid clearance of viral particles from the circulation by the liver support the possibility of active replication [30,31,32].

When the prevalence of the plasma-associated viruses was determined in individual samples using real-time PCR none showed a statistically higher rate of detection or higher viral loads as determined by Ct when comparing plasma from 13 neurological or 14 respiratory cases to plasma from 41 healthy horses. Only the new eqcopivirus showed a trend toward a higher rate of detection in plasma from unexplained respiratory cases (30% versus 17%) (*p* = 0.443). CSF and respiratory swabs where available only from neurological or respiratory cases respectively. The rate of virus detection could therefore not be measured in these anatomical compartments in healthy animals. Horse parvovirus-CSF and eqcopivirus were also detected in 1/13 (different) CSF samples and in 3/13 (different) respiratory swabs each. The initial characterization of horse parvovirus-CSF genome was also from a CSF sample from a different unexplained neurological case [20].

PCR previously showed EqPV-H DNA to be present in the plasma of 13% healthy horses in the US [2] and 12% of healthy race horses in China [13], rates of viremia similar to that detected here in 6/41 (15%) of healthy horses. 

The *Parvoviridae* family consists of non-enveloped, icosahedral, viruses with single stranded DNA genomes of 4 to 6 Kb [27,28]. Eight ICTV approved genera, including *Copiparvovirus*, are currently included in the *Parvoviridae* family [33]. The eqcopivirus genome described here represents the third parvovirus species confirmed to infect horses by virtue of detection in multiple equine plasma, CSF, and respiratory swab samples. That all parvoviruses identified to date in horses belong only to the copiparvovirus genus is somewhat unexpected and future studies are likely to expand the list of known equine parvoviruses. While a higher rate of eqcopivirus DNA detection was found in the plasma of horses with unexplained respiratory signs (30%) versus plasma from healthy horses (17%) that difference, based on the limited number of samples tested, was not statistically significant. Because CSF and respiratory swabs from healthy animals were not available only PCR results from plasma could be compared for disease association. Viruses resulting in neurological and respiratory disease may be expected to be present in CSF or respiratory fluids respectively as were both horse parvovirus-CSF and eqcopivirus. The collection of CSF and respiratory swabs samples from healthy animals may be necessary to better test for possible associations between these copiparvoviruses and unexplained neurological and respiratory signs. The now repeated detection of horse parvovirus-CSF [20] (*n* = 2) and eqcopivirus (*n* = 1) in CSF of horses indicates a possible role for these viruses in these neurological signs which will require further studies. The availability of eqcopivirus genome sequences will now also allow the design of hybridization probes to determine whether infected cells can be identified in fixed brain or lung tissues from animals with unexplained neurological or respiratory signs.

## Figures and Tables

**Table 1:** Distribution of translated sequence reads with similarity E score of <10^−10^ to known mammalian viral proteins.

Disease and Sample Types	Total Reads	Equine Hepacivirus	Equine Pegivirus D	Equid Gamma Herpesvirus2	Equid Gammaherpes Virus5	Horse Parvovirus-CSF	Novel Parvovirus: Eqcopivirus	Bosavirus
Neuro Pool1 Plasma	2,575,048					1023	17,058	
Neuro Pool4 CSF	1,106,824							
Neuro Pool2 Plasma	1,612,796	7						
Neuro Pool5 CSF	1,028,398							
Neuro Pool3 Plasma	2,080,966							
Neuro Pool6 CSF	1,057,992							
Respiratory Pool7 Plasma	1,509,792		317				36,069	
Respiratory Pool10 Swab	622,376							1719
Respiratory Pool8 Plasma	603,470					1087	16,355	
Respiratory Pool11 Swab	949,260			120	25			880
Respiratory Pool9 Plasma	1,520,736							
Respiratory Pool12 Swab	629,274							395

**Table 2:** Real-time PCR results and Ct values. Within each of the 2 clinical groups different samples from the same animals are labeled with the same number. Lowest to highest Ct labeled red to white.

	Sample Type	Pool	Equine Hepacivirus	EqPV-H	Eqcopivirus	Horse Parvovirus-CSF	Bosavirus
**Neurological Signs**	Plasma 1	Pool1			30.32		
	Plasma 2						
	Plasma 3						
	Plasma 4		27.7			20	
	Plasma 5	Pool2					
	Plasma 6		34.65	36.73			
	Plasma 7		23.6				
	Plasma 8						
	Plasma 9	Pool3					
	Plasma 10			33.82			
	Plasma 11						
	Plasma 12						
	Plasma 13						
	CSF 1	Pool4					
	CSF 2						
	CSF 3						
	CSF 4						
	CSF 5	Pool5					
	CSF 6						
	CSF 7						
	CSF 8						
	CSF 9	Pool6				30	
	CSF 10						
	CSF 11						
	CSF 12				36.5		
	CSF 13						
**Respiratory Signs**	Plasma 1	Pool7		37.78			
	Plasma 2						
	Plasma 3						
	Plasma 4				34		
	Plasma 5				27.53		
	Plasma 6	Pool8					
	Plasma 7				30.16		
	Plasma 8						
	Plasma 9			34.6	34.01	21	
	Plasma 10	Pool9					
	Plasma 11						
	Plasma 12						
	Plasma 13						
	Plasma 14						
	Swab 1	Pool10					26.85
	Swab 2						25.65
	Swab 3		25.28				26.4
	Swab 4				36.7		26.82
	Swab 6	Pool11					25.02
	Swab 7				32.62		27.75
	Swab 8						25.57
	Swab 9						26.24
	Swab 10	Pool12				21.2	24.92
	Swab 11					23.1	23.37
	Swab 12						26.63
	Swab 13				32.41		23.29
	Swab 14		37.2			26	25.3
**Healthy Control Group**	Plasma 1						
	Plasma 2					16.8	
	Plasma 3		21.8	37.3			
	Plasma 4		26.5				
	Plasma 5				36.5		
	Plasma 6						
	Plasma 7						
	Plasma 8						
	Plasma 9						
	Plasma 10						
	Plasma 11				28.08		
	Plasma 12						
	Plasma 13						
	Plasma 14				30.21		
	Plasma 15						
	Plasma 16						
	Plasma 17		26.08				
	Plasma 18						
	Plasma 19		23.02	36.9			
	Plasma 20						
	Plasma 21						
	Plasma 22						
	Plasma 23		23.53				
	Plasma 24						
	Plasma 25		25.11				
	Plasma 26						
	Plasma 27			33.83			
	Plasma 28						
	Plasma 29						
	Plasma 30						
	Plasma 31			33.99	30.09		
	Plasma 32			37.93	26.34	30.1	
	Plasma 33						
	Plasma 34						
	Plasma 35						
	Plasma 36			33.22			
	Plasma 37						
	Plasma 38						
	Plasma 39						
	Plasma 40			34.69	28.63		
	Plasma 41				30.12		

**Table 3:** Rate of real-time PCR detection for equine viruses and association with unexplained neurological or respiratory diseases using Fisher’s exact test.

	Neuro Plasma *n* = 13	Neuro CSF *n* = 13	Respiratory Plasma *n* = 14	Respiratory Swab *n* = 13	Healthy Control Plasma *n* = 41	Total Samples *n* = 94	*P* Value = Plasma Neurological Versus Healthy	*P* Value = Plasma Respiratory Versus Healthy
Equine flavivirus	3 (23%)	0	0	2 (15%)	6 (15%)	11 (12%)	0.32	0.67
EqPV-H	2 (15%)	0	2 (15%)	0	7 (17%)	11 (12%)	1	1
Eqcopivirus	1 (7%)	1 (7%)	4 (30%)	3 (23%)	7 (17%)	15 (16%)	0.443	0.663
Horse parvovirus-CSF	1 (7%)	1 (7%)	1 (7%)	3 (23%)	2 (5%)	8 (8.5%)	1	1

## Supplementary Materials

The following are available online at https://www.mdpi.com/1999-4915/11/10/942/s1, Table S1: Real time PCR conditions.
